# Editorial: Women in veterinary epidemiology and economics

**DOI:** 10.3389/fvets.2023.1212004

**Published:** 2023-05-22

**Authors:** Alejandra Victoria Capozzo, Flavie Vial

**Affiliations:** ^1^Institute of Virology and Technical Innovations, National Research Council (CONICET), National Institute of Agricultural Technologies (INTA), Buenos Aires, Argentina; ^2^Animal and Plant Health Agency, Addlestone, United Kingdom

**Keywords:** veterinary, gender, equality, authorship, research

While the number of women graduating from veterinary schools has increased globally over the last few decades, this has not translated into reduced gender bias and inequity in academia and veterinary science research (1). Gender-based discrimination starts at university where women veterinary students are pushed toward “women-majority fields” (e.g., small animal medicine) (2) or where they face discrimination during animal husbandry placements (3). Following graduation, there is clear evidence that gender differences persist in pay and attainment of senior and leadership positions (4). Women's advancement and standing in academic veterinary medicine may in part be influenced by pronounced gender differences in the authorship of veterinary research articles. Women are less likely to be a senior author on a research paper and they are significantly underrepresented in some fields such as surgical and production animal research (5). Gender disparity in professional leadership roles like editorial boards—the median publisher in veterinary sciences had 27.5% editorships belonging to women (6)—can summate by impairing peer recognition and academic advancement.

Our Research Topic aimed to highlight the diversity of work performed across the entire breadth of Veterinary Epidemiology and Economics by teams in which at least 50% of the researchers identified as women.

We start with a veterinary public health study from Thailand carried out by Singhla and Boonyayatra. Their work not only seeks to estimate the prevalence of bovine tuberculosis in slaughtered animals at the Chiang Mai Municipal abattoir, but also to contrast the sensitivity and specificity of the visual meat inspection procedure vs. identification of *M. bovis* by PCR.

A meta-analysis presented by Khanal et al., also from Thailand, looks at the prevalence of Methicillin-resistant *Staphylococcus aureus* (MRSA) in dairy cattle. MRSA represents a significant zoonotic risk, with a potential of being transmitted not only to dairy farmers but across the dairy supply chain to the wider public. An interesting One Health case study!

Two pieces of research are concerned with management practices to improve animal welfare and herd health in large Hungarian dairy cattle farms. The first by Ózsvári and Ivanyos considers the use of pre- and post-milking teat disinfectants and milking machine cleaning products, and links to udder health, in large commercial Holstein-Friesian farms. The second by Várhidi et al. assesses the use of probiotics in nutrition and herd health management as well as the views and experiences of farm nutrition experts.

The flag of veterinary economics was flown by Jerlström et al.. They use stochastic partial budget analysis to measure the economic impact of two strategies aimed at reducing the prevalence of lung lesions in Swedish pigs.

As well as their important contribution to more traditional veterinary Research Topics, women are pushing boundaries and helping new research fields grow such as outcomes research (Dewsbury et al.). Despite being well-established in human medicine, outcomes research, which entails the application of clinical and population-based methods to optimize healthcare practices and interventions, has only recently started to be explored within the context of animal health and veterinary medicine.

We also want to highlight contributions which are the results of collaborations between female scientists from emerging economies and from leading economies. A team of researchers from Thailand and the US (Boonyayatra et al.) use social networks to describe dairy cattle movements in Northern Thailand and identify highly connected districts which represent key areas for disease transmission, surveillance, and control. A research collaboration spanning Australia and Fiji provide an overview of the long-term Bovine Brucellosis and Tuberculosis Eradication and Control program in Fiji (Argelis Garcia et al.). While important improvements in the program have been noted between 2014 and 2020, enhancements in data capture and harmonization as well as increased farmers compliance are now required to make further progress toward eradication of the diseases. International collaborations could be a positive model to promote gender equality in scholarly authorship, in particular for scientists from regions of the world where men are the most overrepresented academically and professionally.

Two submissions to this Research Topic brought up women's perspective exploring gender balance in the animal production sector and the veterinary profession.

While “women are among the most involved in and served by [farming] co-operative organizations”, they are also “the least likely to hold high-ranking and decision-making roles” (7). Hansen and Asmild discuss the structural, cultural, historical, and institutional barriers limiting women's representation on the boards of farmer-owned cooperatives in Denmark. They invite future research to focus on documenting the impact of having more women on boards on the overall performance of cooperatives.

Finally, Stärk et al. challenge the readers to ask themselves why—despite the feminization of veterinary medicine as a profession—so few women either pursue a career leading to, or are successful at securing a leadership role such as being a Chief Veterinary Officer? Several possible explanations to this “leaky pipeline” are put forward but a thorough intentional examination of the field by its practitioners is required to trigger a systemic change in the veterinary medicine work culture, into one where both public and private organization's recruiting and progression policies truly support gender equality and other forms of diversity.

It is important to note that the gender gap in the authorship of veterinary research articles has improved dramatically over the past 20 years (5). Just over two-third (27) of the 39 authors in this Research Topic identified as women. Eighty percent of the first author and of the last author positions were occupied by female scientists. However, it is equally important to observe that more generally significant disparities persist and that the gender gap does vary across and within various geographies. While gender equity in veterinary sciences cannot solely be assessed by looking at research papers, all of us involved in the scientific production and throughout the publication process itself have role to play to increase the visibility of female role models for young women contemplating careers in academia.

## Author contributions

All authors listed have made a substantial, direct, and intellectual contribution to the work and approved it for publication.
